# Traumatic L5 Posterolateral Spondyloptosis: A Case Report and Review of the Literature

**DOI:** 10.7759/cureus.277

**Published:** 2015-06-14

**Authors:** Brandon C Gabel, Erik Curtis, David Gonda, Joseph Ciacci

**Affiliations:** 1 Neurosurgery, University of California, San Diego; 2 Neurosurgery, Texas Children's Hospital

**Keywords:** trauma, spine, retrolisthesis, spine fractures, spinal cord injury, spondyloptosis, fracture dislocation

## Abstract

Traumatic retrolisthesis of the lumbar spine is a rare clinical entity. Only a few case reports have shown retrolisthesis of the fractured fragment over the inferior vertebral body. Fracture dislocations of the spine are unstable injuries that require operative fixation to restore alignment and prevent progressive deformity.

We present the case of a traumatic L5-S1 fracture dislocation with retrolisthesis of the L5 vertebral body over the superior aspect of S1 managed with anterior, middle, and posterior column reconstruction. The patient presented with paraplegia and bowel and bladder incontinence.

Retrolisthesis fracture dislocations injuries are rare, and as such, there are no guidelines regarding their management. In our case, we performed an L5 vertebrectomy with anterior, middle, and posterior column reconstruction via a posterior approach using a lumbosacral-pelvic construct. The patient did not regain function in his distal lower extremities postoperatively.

## Introduction

Traumatic retrolisthesis of the lumbar spine is a rare clinical entity. There are several case reports that have described significant fracture dislocations of the lumbosacral junction, but only a few have shown retrolisthesis of the fractured fragment over the inferior vertebral body [1-4]. It is not surprising that large force vectors are required to generate these injuries. Fracture dislocations of the spine are unstable injuries that require operative fixation to restore alignment and prevent progressive deformity. We present the case of a traumatic L5-S1 fracture dislocation with retrolisthesis of the L5 vertebral body over the superior aspect of S1 managed with anterior, middle, and posterior column reconstruction.

## Case presentation

The patient was a 27-year-old male who presented after a high-speed motor vehicle accident. On initial neurologic assessment, the patient was noted to have 1/5 hip flexion bilaterally but was otherwise 0/5 strength in the lower extremities. Sensation was present in the anterolateral thighs and otherwise absent in the lower extremities. Rectal tone was flaccid. A Foley catheter was placed as part of his initial trauma workup. He was neurologically intact in the upper extremities.

**Figure 1:**
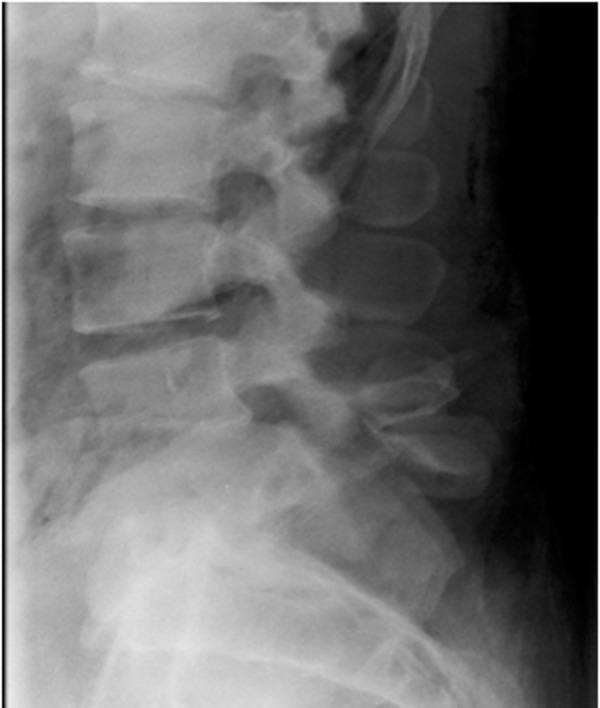
Preoperative lateral x-ray This is a preoperative lateral x-ray demonstrating a fracture dislocation with a posterior retrolisthesis of the L5 vertebrae over S1.

**Figure 2:**
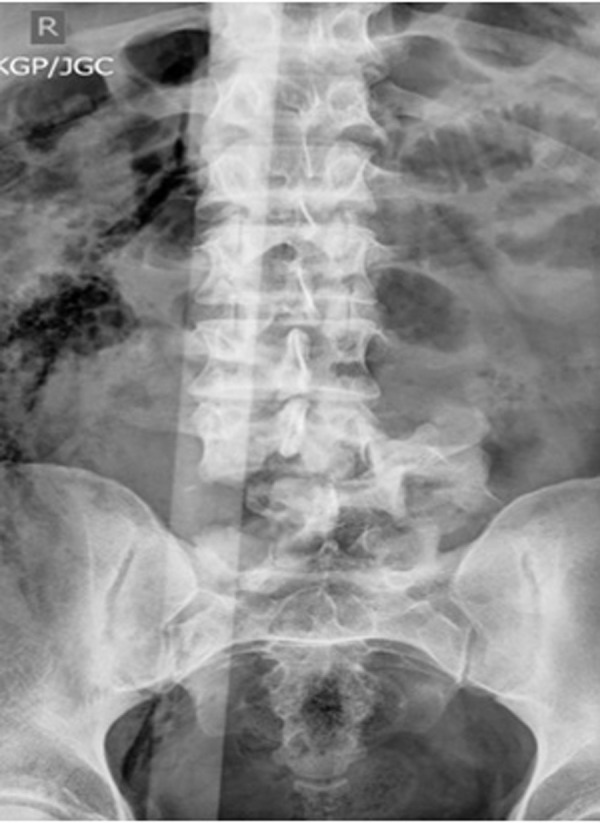
Preoperative anteroposterior x-ray This is a preoperative anteroposterior x-ray demonstrating a fracture dislocation of the L5 vertebrae relative to S1. There is significant lateral listhesis apparent.

**Figure 3:**
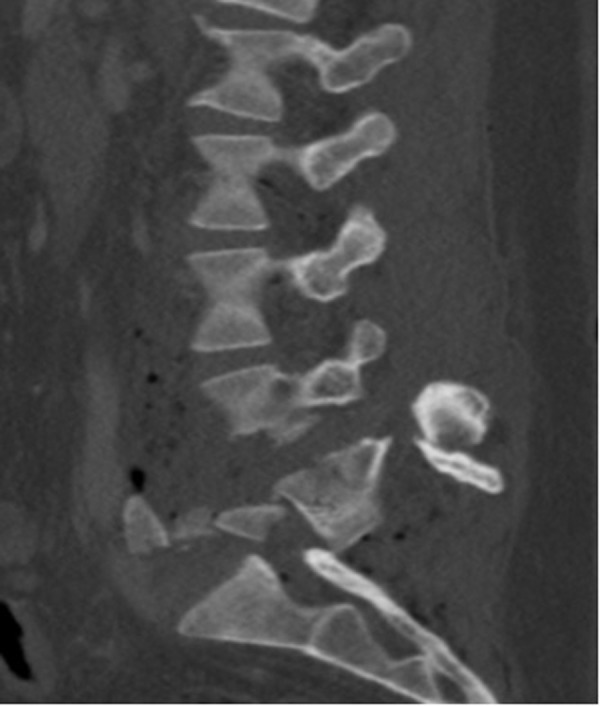
Preoperative parasagittal CT This is a preoperative parasagittal CT demonstrating a fracture dislocation with posterior retrolisthesis of the L5 vertebrae over S1.

**Figure 4:**
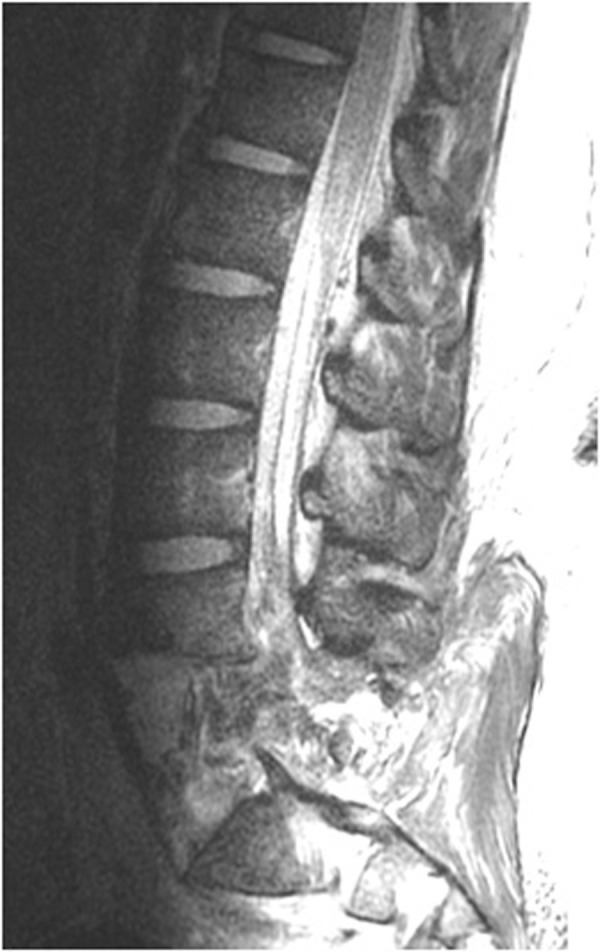
Preoperative parasagittal MRI This is a preoperative parasagittal MRI showing gross ligamentous instability and complete obliteration of the thecal sac at the level of injury.

On arrival, the patient had massive trauma to the right lower quadrant of the abdomen and was taken to the operating room emergently for exploratory laparotomy. After resuscitation, imaging of his neural axis with both CT and MRI revealed a posterolateral retrolisthesis of L5 over S1 with a complete thecal sac obliteration (Figures 1-4). There were additional, less severe fractures at the L3 and L4 vertebral levels. Pneumorachis was also apparent on imaging, which tracked to the C6 and C7 spinal levels. However, there were no additional fractures seen in the thoracic or cervical spine.

**Figure 5:**
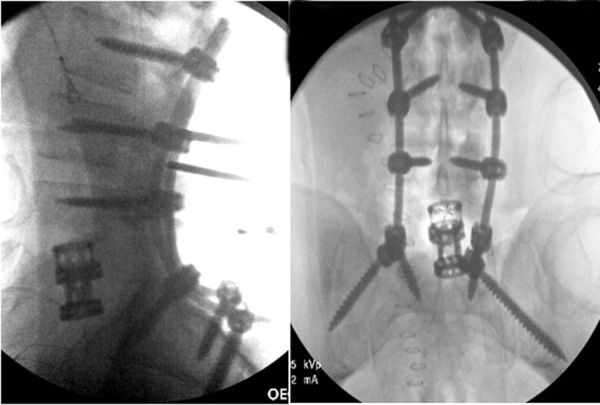
Intraoperative fluoroscopy Intraoperative fluoroscopy showing fixation of the spine from L2 to the sacroiliac joint. An interbody cage was placed at the L5 level to provide anterior column support.

**Figure 6:**
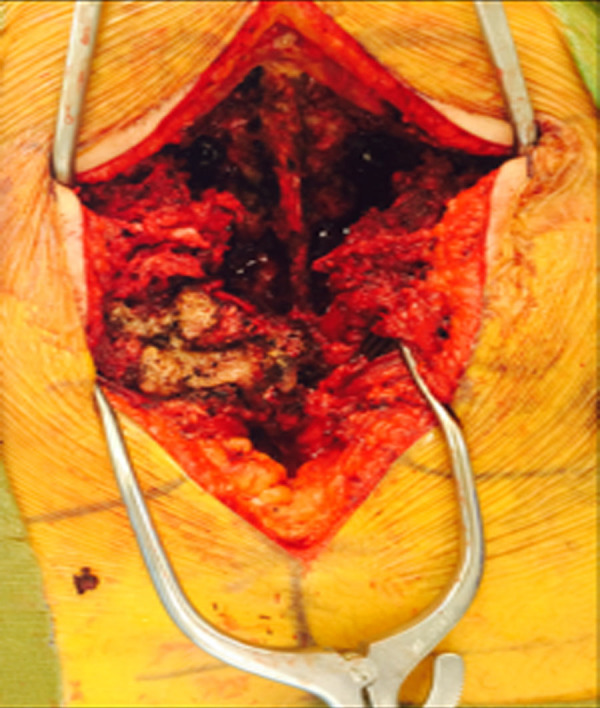
Intraoperative in-situ photograph This is an intraopertaive in-situ photograph of the fractured L5 vertebral body (image left). The spinous processs of the L4 vertebrae is at the top of the image.

**Figure 7:**
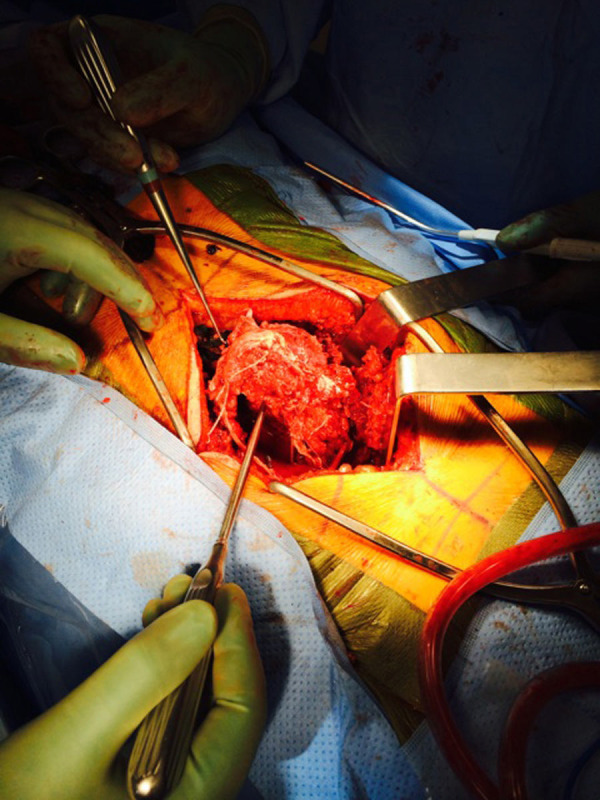
Intraoperative photograph This image shows elevation of the dislocated vertebral body via a posterior incision.

**Figure 8:**
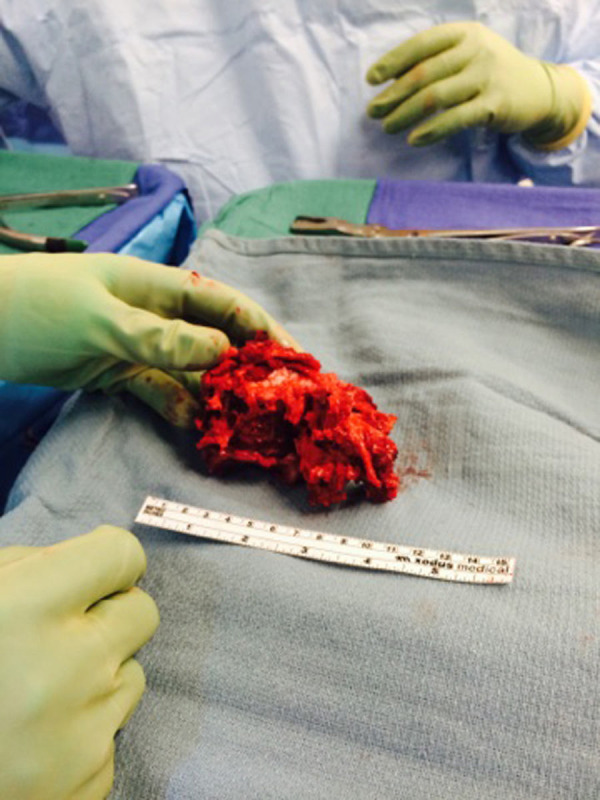
Bony fragment This is a photograph of the vertebral body after en-bloc removal.

He was taken to the operating room non-emergently to address his lumbar spine injuries. The patient was placed on a spinal table without reduction of his fracture. He underwent an L2 to sacroiliac posterior instrumented fusion with L5 vertebrectomy and placement of an interbody cage (Figure 5). The entirety of the case was performed from a posterior approach. The retropulsed L5 vertebral body was readily apparent after opening the posterior lumbosacral fascia (Figures 6-7) and was removed in one piece (Figure 8). Intraoperatively, the dura of the thecal sac had been destroyed, and there was significant nerve root injury apparent.

Postoperatively, the patient continued to have 1/5 strength with hip flexion and maintained sensation in the anterolateral thigh, but otherwise continued to be a functionally complete paraplegic without bowel or bladder control. 

Informed patient consent was obtained for this patient's surgical treatment. No identifying patient information was disclosed in the preparation of this paper nor in the figures noted above.

## Discussion

Most of the studies discussing fracture dislocations of the lumbosacral junction are case reports or small case series [1-4]. These are remarkably uncommon injuries, and in most cases, the L5 vertebral body dislocates anteriorly relative to the sacrum. Aihara, et al. reviewed 57 reported cases, including seven of their own, and noted that in only four cases was the dislocation directed posteriorly [1]. They proposed a classification of these injuries to help guide management, but their system did not include a category for posterior dislocations [1]. As such, surgical management of these injuries remains challenging.

The goal of surgery is to restore anatomical alignment and prevent future deformity. In our particular case, reduction of the fractured L5 body was not feasible, and a vertebrectomy was performed. Corpectomies and/or vertebrectomies at the L5 level are notoriously difficult operations [5]. Cage placement at this level is difficult because of the inherent lordosis of the lumbosacral junction. Most commercially available cages do not have sufficient lordosis to sit flush on the end-plates of S1 and L4. Therefore, anterior reconstruction alone is traditionally thought of as an insufficient means of restoring stability. However, Dai, et al. reported excellent fusion results in non-traumatic L5 corpectomies managed via an anterior only approach [6]. Although their data included non-traumatic lesions, they hypothesized that posterior instrumentation may be unnecessary in select patients. However, the relevance of their data to fracture dislocations is questionable because of the inherent ligamentous instability seen in three-column injuries. In fact, many authors advocate managing L5 burst fractures with posterior instrumentation alone, without anterior column reconstruction, given the inherent difficulties in performing a corpectomy / vertebrectomy at this level [7].

Posterior instrumentation, with or without anterior column reconstruction, has become the “work-horse” for most of the unstable lumbosacral spine injuries. Pedicle screw fixation has been shown to provide superior stability when used in conjunction with anterior instrumentation [8]. Some surgeons advocate sacropelvic fixation to improve fusion rates and prevent distal construct failure; these concerns are especially true in patients with long constructs [9]. Sacropelvic fixation can be accomplished via iliac screws, iliac bars, and/or sacroiliac screws. Sacroiliac screws, also known as modified S2 screws, are being used with increasing frequency. One study showed that sacroiliac screws are biomechanically equivalent to iliac bolts [10]. The use of iliac bolts and/or iliosacral screws has been shown to off-load the forces on S1 pedicle screws by a substantial margin, which may prevent instrumentation failure and improve fusion results.

The lumbosacral pivot point, defined as the point where the L5-S1 disc space meets the middle osteoligamentous column, has important implications when reconstructing the lumbosacral region. During flexion, the pivot point causes the ventral components of L5 and S1 to come closer together, whereas the dorsal elements extend further apart. Since construct rigidity is correlated to fusion, the prevention of significant forces during flexion, extension, lateral bending, and axial rotation are the goal of most spinal fusion procedures [11]. In long constructs, S1 screws by themselves may fail, especially since they absorb a substantial portion of the construct stress. S1 screws obtain most of their purchase via the cancellous bone of the sacrum; S1 screws are also subject to significant flexion force vectors. Both of these factors increase construct failure without concomitant pelvic fixation. McCord, et al. noted that in order to obtain rigidity of long constructs during flexion, pelvic instrumentation should be inserted anterior (ventral) to the lumbosacral pivot point [12]. In other words, without pelvic instrumentation, the significant forces acting on the pivot point during flexion may cause hardware failure resulting in pseudoarthrosis. This is especially relevant when there is co-existent anterior column instability.  

In our case, we removed the posteriorly dislocated L5 vertebrae and reconstructed the anterior column by placing an interbody cage. Given the known difficulties in the surgical management of L5 corpectomies, as well as the significant ligamentous instability of this injury, we elected to perform a concomitant posterior instrumented fusion. We felt that sacroiliac screws, in conjunction with L2, L3, L4, and S1 pedicle screws would provide sufficient support to our anterior construct by preventing failure during flexion; we felt this construct would give the patient the best chance of long-term fusion.

Other important considerations in this case include ruling out occult non-contiguous fractures. It is well documented that occult fractures may be present at non-contiguous spinal levels [13]. We routinely obtain CT and, when indicated, MRI imaging of the entire spinal axis in severe fracture dislocations. It is also important to address life-threatening injuries. Significant intra-abdominal injuries, including injuries to the aorta, vena cavae, iliac veins, and/or arteries can be life-threatening and should be dealt with prior to mechanical stabilization of the spine. Additionally, bony fragments may tamponade occult vascular injuries and should be dealt with cautiously during surgical management of the spinal fracture. 

## Conclusions

Traumatic fracture dislocations of the L5 vertebral level are rare injuries. They are inherently unstable fractures that require surgery to restore anatomic alignment and prevent deformity. There is no standard treatment paradigm for these injuries. As such, the type of surgical procedure performed depends on the unique circumstances of each individual fracture. We agree with most authors that when a vertebrectomy / corpectomy is performed at this level, a posterior instrumented fusion should accompany the anterior column cage reconstruction to aid fusion. Extending the posterior fusion to the sacropelvic junction may provide additional support in severe cases.
